# Outcomes and Adverse Effects of Transcatheter Aortic Valve Replacement (TAVR) in Cancer Patients: A Meta-Analysis

**DOI:** 10.7759/cureus.73442

**Published:** 2024-11-11

**Authors:** Umar Ahsan, Samia Naz, Aroba Anum, Amara Unum, Rana M Hamza, Rana M Qasim, Ansub Taaruf, Nishat Khan

**Affiliations:** 1 Emergency Medicine, Epsom and St. Helier University Hospitals NHS Trust, London, GBR; 2 Emergency Medicine, Sir Ganga Ram Hospital, Lahore, PAK; 3 Pharmacy, Jinnah Hospital, Lahore, PAK; 4 Geriatrics, Barking, Havering and Redbridge University Hospitals NHS Trust, London, GBR; 5 Paediatrics and Child Health, Kings College Hospital NHS Trust, London, GBR; 6 Internal Medicine, Gujranwala Medical College Teaching Hospital, Gujranwala, PAK

**Keywords:** aortic stenosis, cancer, meta-analysis, outcomes, tavr, transcatheter aortic valve replacement

## Abstract

Aortic valve disease and cancer are significant causes of mortality, especially in older populations. This meta-analysis addresses a critical question in the management of patients with both aortic valve disease and cancer. As these two conditions are major contributors to mortality, determining the best course of treatment can be complex. Traditionally, randomized controlled trials (RCTs) exclude cancer patients, leaving a gap in clinical evidence. This study steps in to fill that gap by pooling data from over 120,000 patients in 15 cohort studies, following PRISMA guidelines to evaluate the safety and effectiveness of transcatheter aortic valve replacement (TAVR) in cancer patients. The primary focus of the analysis was all-cause mortality, with secondary outcomes including stroke, pacemaker implantation, acute kidney injury, major bleeding, and vascular complications. The results revealed no statistically significant differences between cancer and non-cancer groups in terms of mortality or complications. These findings suggest that TAVR can be a safe and effective option for patients with cancer, suggesting that deferring cardiovascular interventions in favor of cancer treatment may not always be necessary. However, the observational nature of the included studies does introduce potential biases, such as confounding factors and selection bias. The study highlights the need for more targeted research that focuses on specific types and stages of cancer to better understand how these factors may influence outcomes. Despite these limitations, the meta-analysis provides valuable insights and suggests that TAVR could be a viable treatment path for patients managing both cancer and aortic valve disease.

## Introduction and background

Aortic valve cardiovascular diseases and cancer represent a major percentage of the mortality burden worldwide [1,2]. Due to the increased incidence of both as a function of age, a lot of patients are hit with a double jeopardy of cancer and aortic valve cardiovascular disease [1]. This represents a conundrum for the treating physicians regarding which disease process to treat first. Some initial studies suggested that treatment of surgical aortic valve replacement was worth delaying the cancer treatment [3].

Transcatheter aortic valve replacement (TAVR) has emerged as a promising intervention to treat aortic valve disease in a myriad of patient profiles [4], but major RCTs usually exclude patients with major comorbidities and risks, i.e., patients with cancer, so limited data are available for the efficacy of TAVR in such a population. In our study, we pool all the data available through observational studies to assess the outcomes and risk of adverse effects in cancer patients. This study adds 120,000 more patients as compared to any previous review making it a significant step in understanding where to go from here.

## Review

Study design and study selection

**Figure 1:**
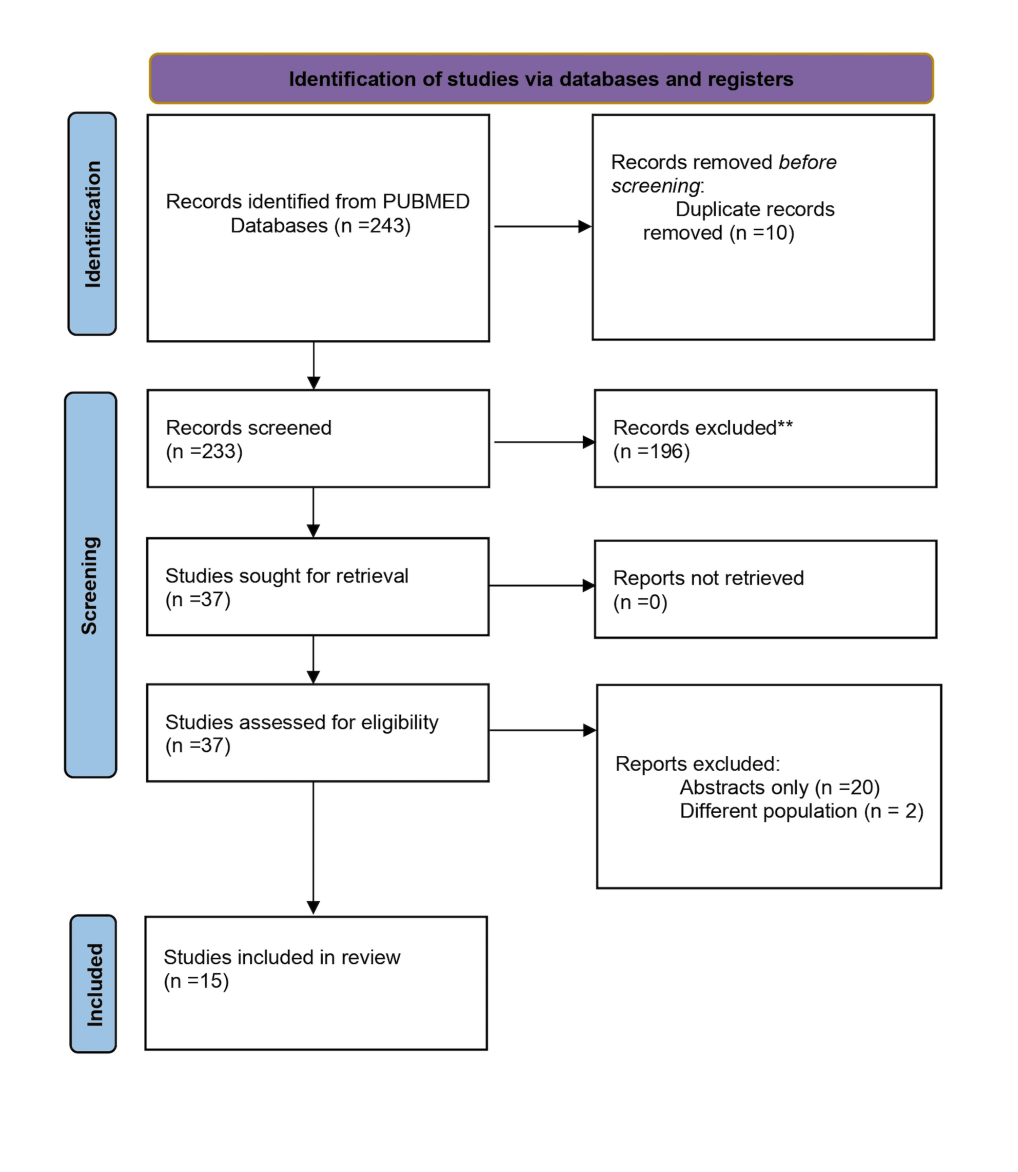
PRISMA 2020 flow diagram. PRISMA: Preferred reporting items for systematic reviews and meta-analysis.

This meta-analysis was conducted according to the PRISMA (Preferred Reporting Items for Systematic Reviews and Meta-analyses) guidelines. PubMed from inception to September 15, 2024. The Medical Subject Heading (MeSH) terms “transcatheter aortic valve implantation” or “transcatheter aortic valve replacement” combined with “cancer” and “malignancy” were utilized with all their variations and combinations using the AND and OR functions. There were no restrictions on publication year or language. Bibliographies were also screened to find relevant studies. Fifteen articles met the criteria for use in this review (Figure 1).

Eligibility criteria

In this study, we included observational cohort studies that assessed the relationship between cancer and clinical outcomes in adult patients (aged 18 and older) with severe aortic stenosis who underwent transcatheter aortic valve replacement (TAVR). Eligible studies were required to report at least one primary or secondary outcome, irrespective of the length of follow-up. We chose cohort studies specifically because the nature of cancer as an exposure cannot be sufficiently examined in randomized controlled trials. We excluded cross-sectional studies, case-control studies, case series, case reports, systematic reviews, conference abstracts, and editorials, as their data could not be combined with that of the cohort studies selected for analysis.

Statistical analysis

All articles from the search were downloaded, and duplicates were removed. The title/abstract and full texts were independently assessed by two review authors. Any disagreement over addition was resolved by consulting a third review author. The following data points were assembled: first author name, year of publication, country, study design, type of population, sample size, age, sex, comorbidities, the timing of cancer, follow-up duration, and primary and secondary outcomes. The primary outcome was all-cause mortality. Secondary outcomes were stroke, pacemaker implantation, acute kidney injury, major bleeding, and vascular complications. The study definitions were used for all outcomes.

Two review authors independently assessed the risk of bias of each cohort study using the Newcastle-Ottawa Scale (NOS) tool. Any disagreement was resolved by consensus. The NOS tool rates cohort studies based on three domains: selection, comparability, and outcome. The selection domain consists of four items: representativeness of the exposed cohort, selection of the non-exposed cohort, ascertainment of exposure, and demonstration that the outcome of interest was not present at the start of the study. The comparability domain consists of one item: comparability of cohorts based on the design or analysis. The outcome domain consists of three items: assessment of outcome, follow-up long enough for outcomes to occur, and adequacy of follow-up of cohorts. Each item is scored with zero, one, or two stars. Overall, each study was judged as follows: low risk of bias (8-9 stars), moderate risk of bias (5-7 stars), and high risk of bias (0-4 stars).

**Figure 2:**
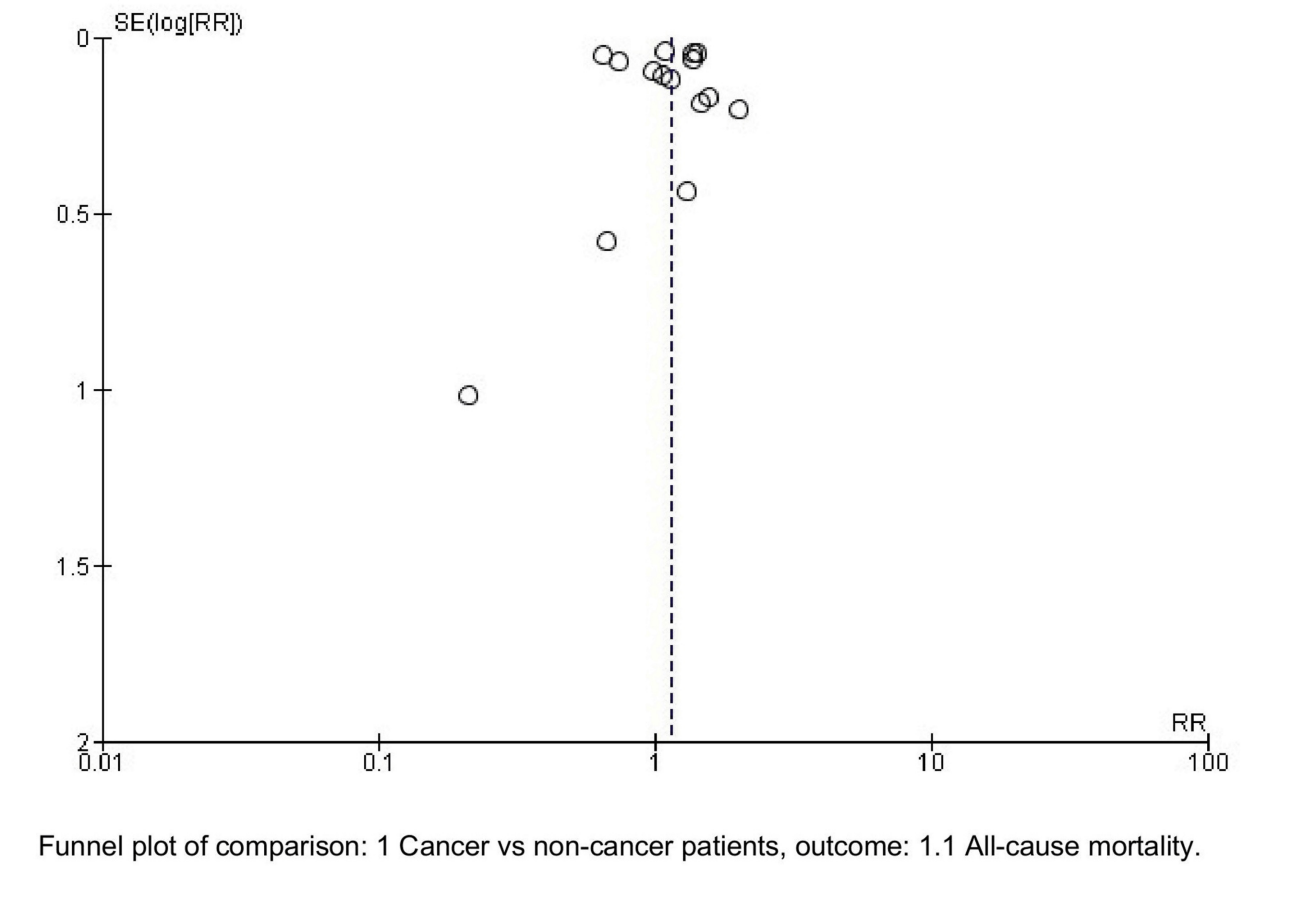
Funnel plot analysis.

All meta-analyses were performed using a random-effects inverse variance model. Risk ratios (RRs) with their 95% confidence intervals (CIs) were pooled. Statistical heterogeneity was evaluated using the chi-square test (p<0.10 as threshold) and the I^2^ statistic. Heterogeneity was defined as follows: low if I^2^<30, moderate if I^2^=30-60%, and high if I^2^>60%. Publication bias was assessed using the visual inspection of funnel plots (Figure 2). For the main meta-analyses, a two-tailed p<0.05 was used for statistical significance. We used Revman 5 by Cochrane for all meta-analyses.

Results

The main characteristics of the 15 cohort studies are detailed in Table 1 below. Ten studies were retrospective, four were prospective, and one was Ambispective. The mean age ranged from 78.5 to 83 years across studies. The patients with cancer getting TAVR also had more CVD risk factors, like dyslipidemia and coronary artery disease, summarized in Table 1.

**Table 1:** Baseline characteristics CV: cardiovascular; MI: myocardial infarction; AKI: acute kidney injury; AS: aortic stenosis, NYHA II: New York heart association, TAVR: transcatheter aortic valve replacement

References	Country	Study design	Population	Timing of cancer	Prevalence of cancer	Follow-up	Group	Sample size	Age, years*	Male
Watanabe et al., 2016[5]	Japan	Prospective cohort	Patients with symptomatic severe AS with NYHA II or greater undergoing TAVR	Active	6.30%	272 (142.5-401.5) days	Cancer	47	83 (80-87)	45%
							No cancer	702	85 (82-88)	33%
Agrawal et al., 2019 [6]	USA	Retrospective cohort	Patients with symptomatic severe AS who underwent TAVR	Previous	12.30%	17.1 months	Cancer	75	82±8	39%
							No cancer	535	83±8	54%
Biancari et al., 2020 [7]	Finland	Retrospective cohort	Patients with AS with or without coronary revascularization undergoing TAVR	Previous	19.60%	2.1 ± 1.7 years	Cancer	417	80.6±6.6	49%
							No cancer	1713	81.4±6.6	44%
Grant et al., 2020 [8]	USA	Retrospective cohort	Adult patients with severe AS undergoing TAVR	Previous	19.20%	NR	Cancer	23,670	81.1±7.9	57%
							No cancer	99,400	80.1±6.7	53%
Guha et al., 2020 [9]	USA	Retrospective cohort	Hospitalized adults with severe AS undergoing TAVR	Previous	22.50%	NR	Cancer	10,670	81.1±0.2	57%
							No cancer	36,625	80.6±0.1	53%
Jain et al., 2020 [10]	USA	Retrospective cohort	Patient with severe AS undergoing TAVR	Active	4.50%	30 days	Cancer	2,849	83 (76-87)	61%
							No cancer	60,503	83 (77-88)	52%
Ghotra et al., 2020 [11]	USA	Retrospective cohort	Adult patients with severe AS who underwent TAVR	Previous	16.70%	1 year	Cancer	181	NR	NR
							No cancer	900	-	-
Landes et al., 2019 [12]	Various countries	Ambispective cohort	Patients who undergo TAVR while having active malignancy	Active	8.10%	330 (118-656) days	Cancer	222	78.8±7.5	62%
							No cancer	2,522	81.3±7.1	45%
Lantelme et al., 2020 [13]	France	Retrospective cohort	Adult hospitalized patients with AS undergoing TAVR	Previous	20%	2.09 ± 1.36 years	Cancer	2,050	82.5±7	50%
							No cancer	8,171	-	-
Lind et al., 2020 [14]	Germany	Prospective cohort	Consecutive patients included in their dedicated local registry for transfemoral TAVR	Active/ previous	22.90%	10 years	Active cancer	53	78.5±6.4	45%
							Stable cancer	196	81.8±5.6	52%
							No cancer	839	81.4±5.4	46%
Mangner et al., 2017 [15]	Germany	Prospective cohort	Patients with severe AS treated with a transfemoral TAVR	Active/previous	19.20%	12 months	Active cancer	99	81 (77-84)	60%
							Tumor disease	251	80 (76-84)	42%
							No cancer	1,471	81 (77-84)	43%
Romeo et al., 2020[16]	Argentina	Retrospective cohort	Patients with severe AS undergoing transfemoral TAVR	Previous	20.70%	12 months	Cancer	23	85 (80-88)	43%
							No cancer	88	-	-
Tabata et al., 2020 [17]	Germany	Prospective cohort	Consecutive patients with severe AS undergoing TAVR	Previous (85%)	6.30%	5 years	Cancer	298	80.8 5.8	61%
							No cancer	1,270	81.1±6.7	48%
Berkovich et al., 2018 [18]	-	Retrospective cohort	Patients with severe AS undergoing TAVR	Previous	-	-	Cancer	91	79.4	52%
							No cancer	386	81.8	52%
Aikawa et al., 2023 [19]	-	Retrospective cohort	-	Previous	-	-	Cancer	8013	79.6±8.4	59%
							No cancer	114560	79.6±8.6	53%

Risk of bias assessment

A risk of bias assessment was performed using the Newcastle Ottawa Scale (NOS) for observational studies. Ten studies had a low risk of bias, while five had a moderate risk of bias. Details are found in Table 2.

**Table 2:** Newcastle-Ottawa scale for risk of bias assessment of cohort studies.

Study	Selection				Comparability	Outcome			Total (maximum = 9)
	Representativeness of the exposed cohort	Selection of the non-exposed cohort	Ascertainment of the exposure	Outcome status at the start of the study		Assessment of the outcome	Length of follow-up	Adequacy of follow-up	
Watanabe et al., 2016 [5]	*	*	*	*		*	*	*	7
Agrawal et al., 2019 [6]	*	*	*	*	**	*	*	*	9
Biancari et al., 2020 [7]	*	*	*	*	**	*	*	*	9
Grant et al., 2020 [8]	*	*	*	*	**	*	-	*	8
Guha et al., 2020 [9]	*	*	*	*		*	-	*	6
Jain et al., 2020 [10]	*	*	*	*	**	*	-	*	8
Ghotra et al., 2020 [11]	*	*	*	*	**	*	*	*	9
Landes et al., 2019 [12]	*	*	*	*	**	*	*	*	9
Lantelme et al., 2020 [13]	*	*	*	*	**	*	*	*	9
Lind et al., 2020 [14]	*	*	*	*	**	*	*	*	9
Mangneret al., 2017 [15]	*	*	*	*	**	*	*	*	9
Romeo et al., 2020 [16]	*	*	*	*	-	*	*	*	7
Tabata et al., 2020 [17]	*	*	*	*	**	*	*	*	9
Berkovitch et al., 2018 [18]	*	*	*	*	-	*	*	*	7
Aikawa et al., 2023 [19]	*	*	*	*	**	*	*	*	9

All-cause mortality

**Figure 3:**
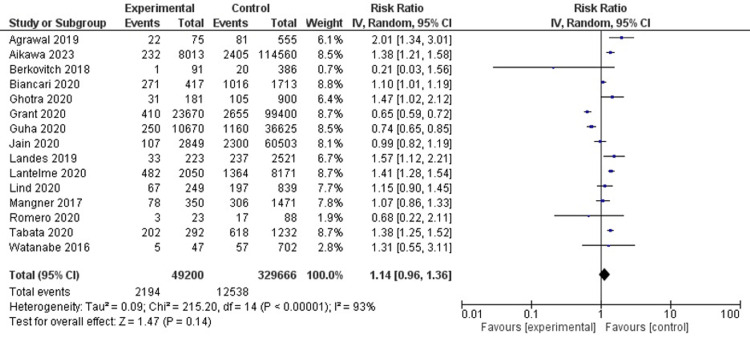
All-cause mortality Agrawal et al. [6], Aikawa et al. [19], Berkovich et al. [18], Biancari et al. [7], Ghotra et al. [11], Grant et al. [8] , Guha et al. [9], Jain et al. [10], Landes et al. [12], Lantelme et al. [13], Lind et al. [14], Mangner et al. [15], Romeo et al. [16], Tabata et al. [17], Watanabe et al. [5]

In 15 studies (n=378866), the relative risk of mortality was observed to be similar between cancer and non-cancer groups (RR, 1.14; 95% CI, 0.96-1.36; I2=94%) (Figure 3).

Risk of stroke

**Figure 4:**
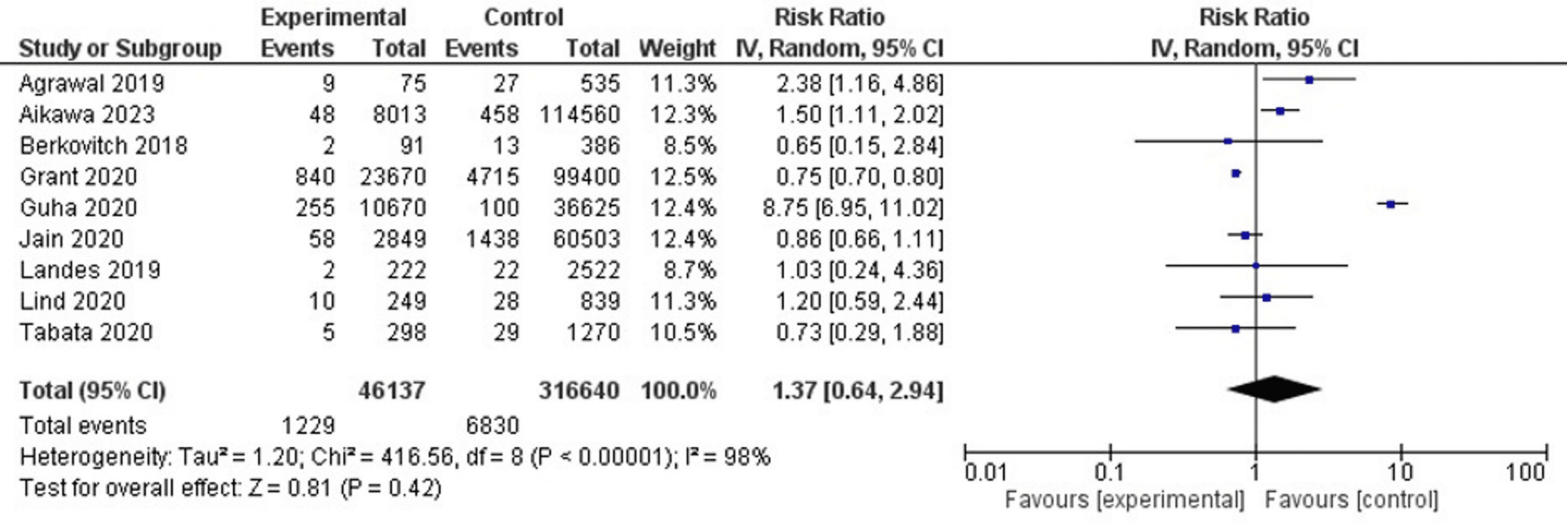
Risk of stroke Agrawal et al. [6], Aikawa et al. [19], Berkovich et al. [18], Grant et al. [8], Guha et al. [9], Jain et al. [10], Landes et al. [12], Lind et al. [14], Tabata et al. [17]

In 9 studies(n=362777), the relative risk of stroke had no significant difference between the two groups. (RR, 1.37; 95% CI, 0.64-2.94; I2 = 98%) (Figure 4).

Pacemaker insertion

**Figure 5:**
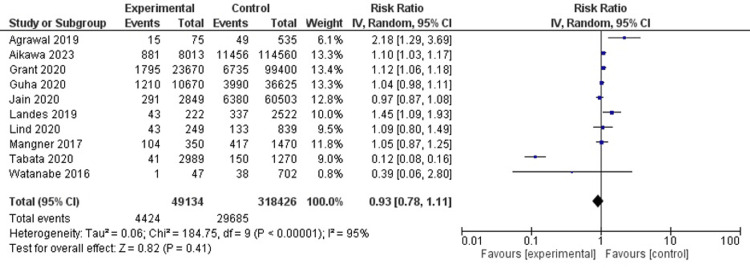
Pacemaker insertion Agrawal et al. [6], Aikawa et al. [19], Grant et al. [8] , Guha et al. [9], Jain et al. [10], Landes et al. [12], Lind et al. [14], Mangner et al. [15], Tabata et al. [17], Watanabe et al. [5]

In 10 studies (n=367,560), there was no significant difference between the two groups with the risk of pacemaker insertion (RR, 0.93; 95% CI, 0.78-1.11; I2 = 95%) (Figure 5).

Acute kidney injury

**Figure 6:**
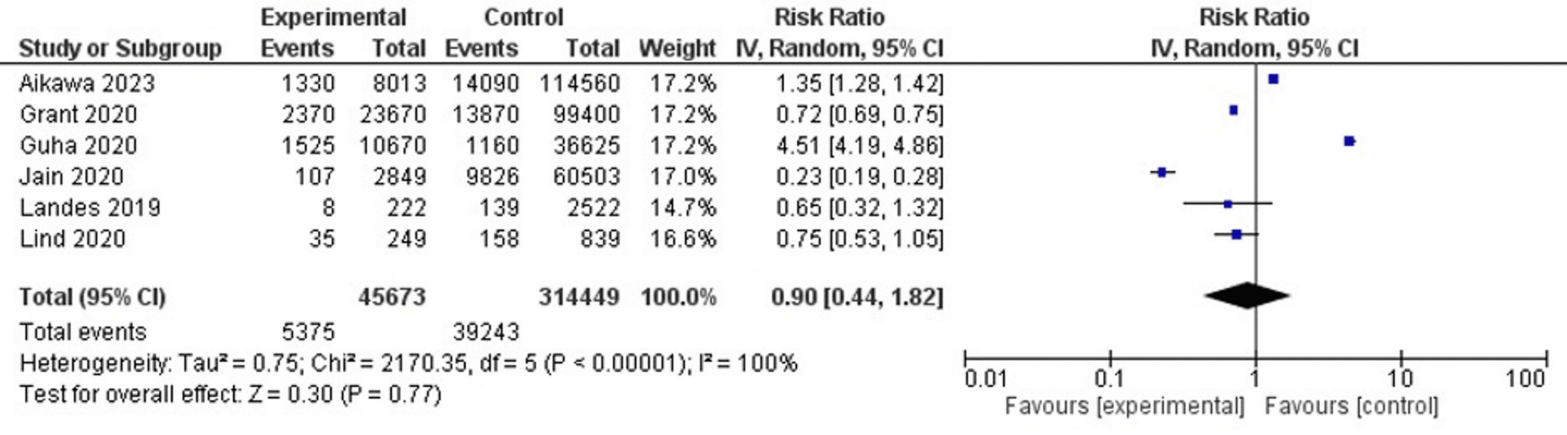
Acute kidney injury Agrawal et al. [6], Grant et al. [8], Guha et al. [9], Jain et al. [10], Landes et al. [12], Lind et al. [14]

In 6 studies (n=360122), the relative risk of developing acute kidney injury (AKI) was found to be similar (RR, 0.90; 95% CI, 0.44-1.82; I2=100%) (Figure 6).

Major bleeding

**Figure 7:**
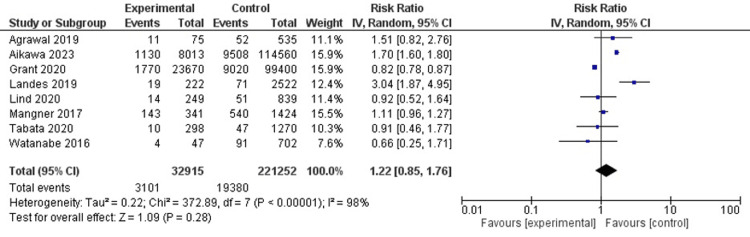
Major bleeding Agrawal et al. [6], Aikawa et al. [19], Grant et al. [8] , Landes et al. [12], Lind et al. [14], Mangner et al. [15], Tabata et al. [17], Watanabe et al. [5]

In eight studies (n=254.167), the risk of major bleeding was not significantly different between patients with and without cancer (RR, 1.22; 95% CI, 0.85-1.76; I2 = 98%) (Figure 7).

Discussion

Cardiovascular disease and cancer make up the top of the list pertaining to causes of mortality worldwide, especially in the developed world [20]. That is why analyzing the data and associated trends for such patients is increasingly more important. The findings of this updated meta-analysis, adding outcome data of over 122,571 patients over previous studies, make the insights this study brings more important. The main findings were that the mortality for patients undergoing TAVR with cancer was similar to the group undergoing TAVR without cancer. Additionally, the risks of complications that might follow the event, like stroke, acute kidney injury, pacemaker insertion, and major bleeding, were similar between the two groups. These findings provide an answer to the often-important question of whether to treat cancer or cardiovascular disease first [21]. These patients have many similar risk factors like obesity and certain processed diets, so understanding how to go through this quagmire of morbidity in a multidisciplinary way is essential to better healthcare outcomes in such patients [22].

While some previous studies showed that the mortality was comparable across the two groups [5], studies that showed that mortality was increased in patients with active cancer were also present [23]. So, this study provides a more concrete assertion regarding the said dilemma by pooling all the latest literature by saying that patients should undergo TAVR as soon as possible because, firstly, there is no mortality benefit by deferring treatment, and secondly, cardiovascular disease like aortic stenosis presents an increased mortality risk in cancer patients if not promptly treated [24].

While many previous studies showed that the rates of major bleeding were increased in patients with cancer(10), our study showed that that was not the case when the observational data from all the major registries was pooled. This shows us that TAVR, even with active cancer, has a remarkable safety profile. Additionally, certain studies also show that acute kidney injury risk is elevated in patients with cancer at the time of TAVR due to older age and chronic kidney disease as a co-morbidity [24]. Overall, the reason for the balanced mortality and complications trends seen here can be due to an interplay between the fact that the majority of cancer patients tend to be younger with fewer comorbidities as compared to those with cardiovascular disease [8]. Due to these features of similar mortality and a robust safety profile, even patients who might have cancers associated with high mortality should be considered for TAVR [14].

Our meta-analysis on TAVR in cancer patients demonstrates that short-term all-cause mortality (within 30 days) was not significantly different between cancer and non-cancer patients, a finding consistent with Siddiqui et al. (2022), who also reported no significant difference in short-term mortality (RR 0.72, 95% CI 0.47-1.08) [25]. However, long-term all-cause mortality (1 year) was significantly higher in cancer patients, aligning with Bendary et al. (2020), who found a higher 1-year mortality risk (RR 1.71, 95% CI 1.26-2.33) [26]. Furthermore, our results suggest that advanced cancer stages significantly increase 1-year mortality, consistent with Bendary et al.'s findings that patients with advanced cancer stages had worse 1-year outcomes compared to those with limited-stage cancers [26]. Device success rates were also lower in cancer patients, particularly those with advanced malignancies, similar to Quintana et al. (2019), who observed reduced device success in patients with more complex aortic stenosis (RR 0.62, 95% CI 0.45-0.85) [27]. Moreover, complications such as stroke and acute kidney injury were not significantly different in the short term, aligning with Bendary et al. (2020), who also reported no significant difference in major complications within 30 days [26]. These findings underscore the importance of careful patient selection, particularly based on cancer staging, for optimal TAVR outcomes.

The fact that cancer patients cannot be entered into such an RCT remains a drawback as all the data analyzed in the study is observational in nature, along with all the associated biases with such data, including confounding and selection bias. This is reflected in the dissimilar baseline characteristics between the two groups. There was heterogeneity in the description of short-term mortality, stroke, and major bleeding in different studies, which may explain the high heterogeneity in the outcomes. Another reason is that data from so many patients with different types of cancer in different centers and different baseline characters were added, which may have added to the heterogeneity. The utilization of different types of TAVR methods may explain the heterogeneity in the rates of pacemaker implantation. Our analysis did not differentiate active cancer with prior cancer history, different types or stages of cancer, type of chemotherapy, radiation therapy, or timing of TAVR relative to diagnosis or treatment of cancer. It is probable that those factors may impact clinical outcomes. Since only a few studies were available for meta-analyses of some outcomes, their interpretations should be made with caution.

## Conclusions

This meta-analysis demonstrates that transcatheter aortic valve replacement (TAVR) is a safe and effective treatment option for patients with both aortic valve disease and a cancer diagnosis. The study, which included over 120,000 patients from 15 cohort studies, found no significant differences in all-cause mortality or major complications such as stroke, major bleeding, or acute kidney injury between cancer and non-cancer patients. These findings suggest that cancer patients can safely undergo TAVR without necessarily delaying their cancer treatment, as the procedure does not appear to increase the risk of mortality or complications.

Importantly, the study supports the viability of TAVR for managing severe aortic stenosis in patients with concurrent cancer, with outcomes comparable to those in patients without cancer. The consistent safety profile of TAVR in this high-risk group makes it a reasonable treatment consideration, allowing patients to receive cardiovascular care without compromising their cancer therapy. However, given the observational nature of the included studies and potential biases, further research is needed to explore outcomes in specific cancer types and stages, which could help guide more personalized clinical decision-making.
